# Blood Circulating miRNA Pairs as a Robust Signature for Early Detection of Esophageal Cancer

**DOI:** 10.3389/fonc.2021.723779

**Published:** 2021-07-23

**Authors:** Yang Song, Suzhu Zhu, Ning Zhang, Lixin Cheng

**Affiliations:** Shenzhen People’s Hospital, First Affiliated Hospital of Southern University of Science and Technology, Second Clinical Medicine College of Jinan University, Shenzhen, China

**Keywords:** microRNA, biomarker, esophageal cancer (EC), gene pair, diagnosis

## Abstract

Esophageal cancer (EC) is a common malignant tumor in the digestive system which is often diagnosed at the middle and late stages. Noninvasive diagnosis using circulating miRNA as biomarkers enables accurate detection of early-stage EC to reduce mortality. We built a diagnostic signature consisting of four miRNA pairs for the early detection of EC using individualized Pairwise Analysis of Gene Expression (iPAGE). Profiling of miRNA expression identified 496 miRNA pairs with significant relative expression change. Four miRNA pairs consistently selected from LASSO were used to construct the final diagnostic model. The performance of the signature was validated using two independent datasets, yielding both AUCs and PRCs over 0.99. Furthermore, precision, recall, and F-score were also evaluated for clinical application, when a fixed threshold is given, resulting in all the scores are larger than 0.92 in the training set, test set, and two validation sets. Our results suggested that the 4-miRNA signature is a new biomarker for the early diagnosis of patients with EC. The clinical use of this signature would have improved the detection of EC for earlier therapy and more favorite prognosis.

## Introduction

Epidemiological data have indicated that esophageal cancer (EC), a common malignant tumor in the digestive system, is the sixth cause of tumor-related death, and is also accompanied by an increasing incidence and mortality worldwide (1). Every year, over 300,000 people die from EC and the number is up to 150,000 and in China (1). Despite the advances in surgical techniques and chemoradiotherapy strategies have extensively improved the prognosis of EC patients, EC remains a deadly cancer of the gastrointestinal tract. Because of its insidious onset, the diagnosis of EC is usually at an advanced stage. Therefore, finding effective biomarkers for the early diagnosis of EC has great significance.

High-throughput technologies have revolutionized non-invasive diagnosis in medical research by the parallel analysis of thousands of molecules in cells or body fluids, including proteins, microbes, coding and non-coding RNAs, *etc.* (2–6). Non-coding RNAs (ncRNAs) regulate gene transcription and recently are emerging as a novel therapeutic targets and promising biomarkers for disease diagnosis and prognosis (7–12). MicroRNAs (miRNAs) are a type of small and highly conserved non-coding RNAs with 18–25 nucleotides in length (13, 14). miRNAs could broadly inhibit the expression of target messenger RNAs (mRNAs) and affect the fundamental cellular and physiological functions in humans (15). In recent years, numerous studies have indicated that miRNAs play as pivotal regulators in the tumorigenesis, progression, proliferation, and metastasis of various cancers, including EC (16, 17). Despite many miRNAs potentially important to cancers are yet to be characterized, their expression patterns have shown their non-invasive diagnosis ability in detecting and monitoring cancer progression (18).

Several studies have investigated the value of circulating miRNAs as potential biomarkers for the early screening of EC (16). Notably, for the gene transcriptome data, it is usually preprocessed using a series of steps, including background correction, signal normalization, and gene summarization (19–21). For each step, several candidate algorithms are available based on different assumptions of data distribution. For instance, the quantile normalization assumes all samples have identical distribution regardless of the sample heterogeneity and conditions, such as cancer and normal (20). However, this most commonly used assumption only holds true when a small fraction of genes are dysregulated. In fact, a considerable fraction of genes are differently expressed in cancer samples due to the very different expression distribution of genes between the cancer and non-cancer samples (20, 21).

Previously we proposed a feature selection method, individualized Pairwise Analysis of Gene Expression (iPAGE) (22), to reduce the mRNA and lncRNA dimension, which is more suitable for the high dimensional miRNA data due to its high simplicity and efficiency. The relative expression change of a pair of genes are considered and only the gene pairs with significant alterations between the detecting groups are remained for further analysis, instead of the single genes with differential expression. Based on a stringent selection criterion, only a few gene pairs are refined and it benefits a lot for the subsequent step of model construction. Currently, we are using the iPAGE strategy for several directions on the forefront of genetic science to come up with more sophisticated results in terms of methylome and single-cell RNA-seq.

The iPAGE strategy fits miRNA expressions well and it is useful in machine learning where complex number systems determine what the computer “learns” or “knows” (2, 23, 24). In this study, we identified a four-miRNA pair signature for the early diagnosis of EC using iPAGE. The performance of the signature was validated using two independent datasets, and it outperformed the other state-of-art biomarkers in both ROC and PRC.

## Materials and Methods

### miRNA Expression Data

The miRNA expression datasets used in this study were downloaded from the Gene Expression Omnibus (GEO, http://www.ncbi.nlm.nih.gov/geo/) database. Using the keywords “esophageal cancer” and “serum” for human miRNA dataset searching, we obtained three datasets GSE122497, GSE106817, and GSE112264 (16, 25–27). All these three datasets were detected using the 3D-Gene Human miRNA V21_1.0.0 platform (GPL21263). More detailed description for each dataset was listed in **Table 1**. No normalization was carried out and only the raw data were used for miRNA pair selection. For the 6-miRNA signature built by Sudo et al. using miRNA expression values (16), the data were normalized using the Robust Multichip Average (RMA) algorithm (28).

**Table 1:** miRNA microarray data sets used in this study.

Accession	Year	Platform	Sample size	EC	Control
GSE122497	2019	GPL21263	5531	566	4965
GSE106817	2018	GPL21263	2847	88	2759
GSE112264	2019	GPL21263	91	50	41

### Detection of miRNA Pairs

**Figure 1:**
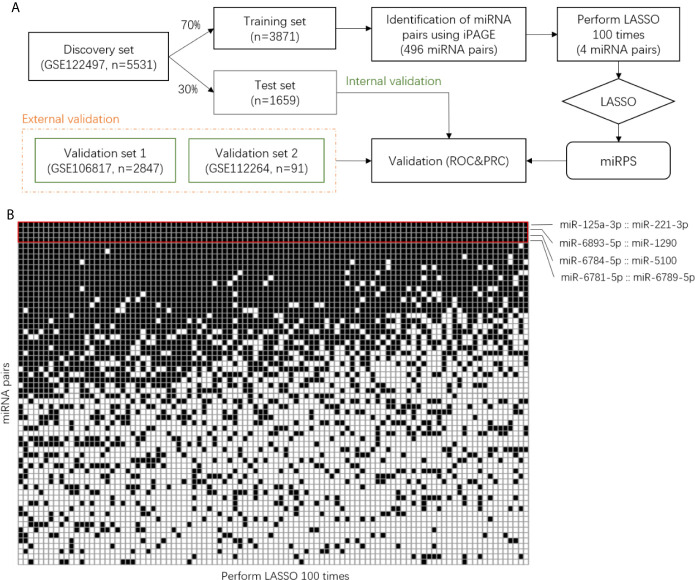
Identification of miRNA pair signature. **(A)** Workflow of this study. **(B)** Binary matrix with rows represent miRNA pairs and columns represent the result of LASSO. The black grid corresponds to the selected pairs. iPAGE, individualized Pair Analysis of Gene Expression. LASSO, Least absolute shrinkage and selection operator.

The dataset GSE122497 contained 566 samples with esophageal squamous cell carcinoma and 4,965 non-cancer samples as controls. 70% of these samples were assigned as the training set and the other 30% samples were set as the test set (**Figure 1A**). Then, the individualized Pairwise Analysis of Gene Expression (iPAGE) strategy was used for feature selection. All possible miRNA pairs were constructed and the reverse pairs with significant relative expression changes were kept for subsequent analysis. The reverse pairs were defined as the expression abundance of the first miRNA consistently larger than the second one in at least 90% of the cancer samples and the first miRNA smaller than the second one in more than 90% of the control samples. In addition to 0.9 defined as the reverse rate, another threshold of 95% was also used for comparison in this study.

### Model Construction

The reverse miRNA pairs selected in the previous step served as candidate markers for the diagnostic signature. Next, these pairs were further refined using least absolute shrinkage and selection operator (LASSO), resulting in a penal of miRNA pairs with assigned coefficients or contribution weights. Hereafter, we named the penal as miRNA pair signature. Considering the results of LASSO are different when set different seeds, we performed LASSO 100 times and utilized the common miRNA pairs to construct the final diagnostic model (**Figure 1A**).

### Performance Evaluation

We evaluated the performance of the miRNA pair signature using both Receiver Operating Characteristic (ROC) curve and Precision-Recall Curves (PRC) on the test set and two independent validation sets, GSE106817 and GSE112264. Measurements of precision, recall, and F-score were also used for evaluation, which were calculated as follows,

Pression=TP/(TP+FP),Recall=TP/(TP+FN),F−score=(2∗Recall∗Precision)/(Recall+Pression),

where TP, TN, FP, and FN denote the number of true positives, true negatives, false positives, and false negatives, respectively. All the above calculations were conducted using R 4.0.3.

## Results

### Data Collection

Circulating miRNAs can be stably detected in serum and serve as potential biomarkers in the non-invasive diagnosis of cancers. To build an effective diagnostic model, we systematically collected the datasets containing miRNA serum samples of Esophageal Cancer (EC) from the GEO database []. Three datasets GSE122497, GSE106817, and GSE112264 were selected using the keywords “esophageal cancer” and “serum”. Using the platform of GPL21263 3D-Gene Human miRNA V21_1.0.0, these three datasets detected 2,565 miRNAs among 8,469 samples, including both EC and control normal samples. GSE122497, containing the highest number of samples (n=5531), was randomly divided into a training set (70%) and a test set (30%). The other two datasets were used as external sets for independent validation, where the larger one GSE106817 with a sample size of 2,847 was defined as validation set 1 and the smaller one GSE112264 (91 samples) was defined as validation set 2.

### Identification of miRNA-Pair Signatures

For the training set, a total of 3,288,330 miRNA pairs composed of 2,565 miRNAs were constructed. We identified 496 miRNA pairs with significant relative expression change, namely, in a pair, the expression values of one miRNA are consistently larger than the other miRNA in at least 90% of the control samples and smaller than the other one in more than 90% of the cancer samples. Then, we selected the miRNA pairs contributing most to the classification using LASSO. Since the resulting pairs were different using the random computation seeds, we carried out LASSO 100 times and determined the miRNA pairs that were consistently selected (**Figure 1B**). Interestingly, a majority of the miRNA pairs were randomly picked up and only four pairs (red boxed) were selected in all the 100 rounds, indicating the importance of these pairs in classification.

**Figure 2:**
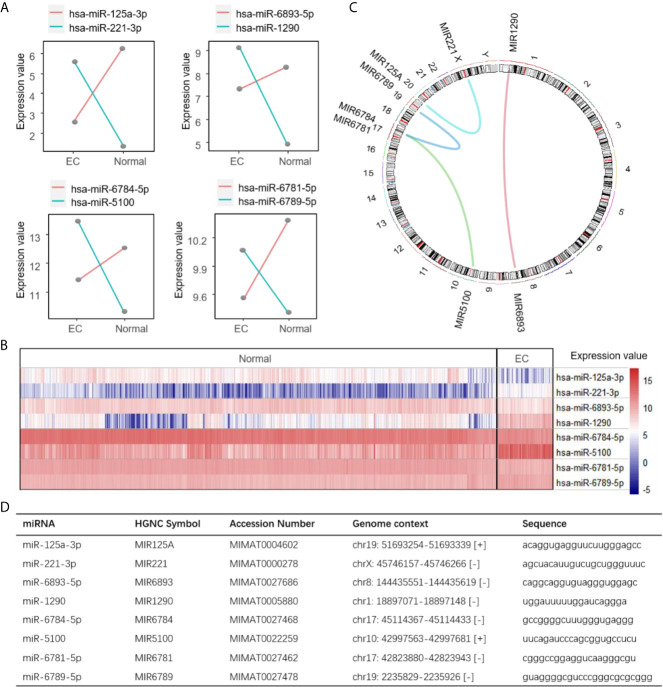
Summarization of the four miRNA pairs. **(A)** Expression values of the four identified miRNA pairs. Line represents the average expression abundance in EC and normal states for a miRNA in the training set. Two lines are intersecting when a pair of miRNAs are reversed in expression between the EC and normal state. **(B)** Heatmap showing the expression value of the four miRNA pairs between EC and normal samples in the training set. **(C)** A circos plot showing the location of the four miRNA pairs in chromosome. Curves in the circle represent the miRNA pairs. **(D)** The genetic information of the miRNA pairs.

Next, we calculated the coefficients of the four miRNA pairs using LASSO to build a risk score, miRPS, reflecting the probability of a patient having EC. The miRPS was calculated as follows: 3.903316 * (hsa-miR-6781-5p, hsa-miR-6789-5p) + 3.613282 * (hsa-miR-6893-5p, hsa-miR-1290) + 3.138672 * (hsa-miR-6784-5p, hsa-miR-5100) + 2.603476 * (hsa-miR-125a-3p, hsa-miR-221-3p) - 8.312100. For each pair, the value is assigned 1 if the expression value of the first miRNA is larger than the second one. Otherwise, it is assigned 0. No coefficient was dominated and the largest one is 3.903316 for the pair of hsa-miR-6781-5p and hsa-miR-6789-5p. The expression value of each miRNA pair was reverse between distinct states (**Figure 2A**). The heatmap illustrates the significant differences of the miRNAs in each pair between cancer and non-cancer samples (**Figure 2B**). We also provided the chromosome and sequence information of the four miRNA pairs for potential further analysis (**Figures 2C, D**).

### Performance Evaluation

**Figure 3:**
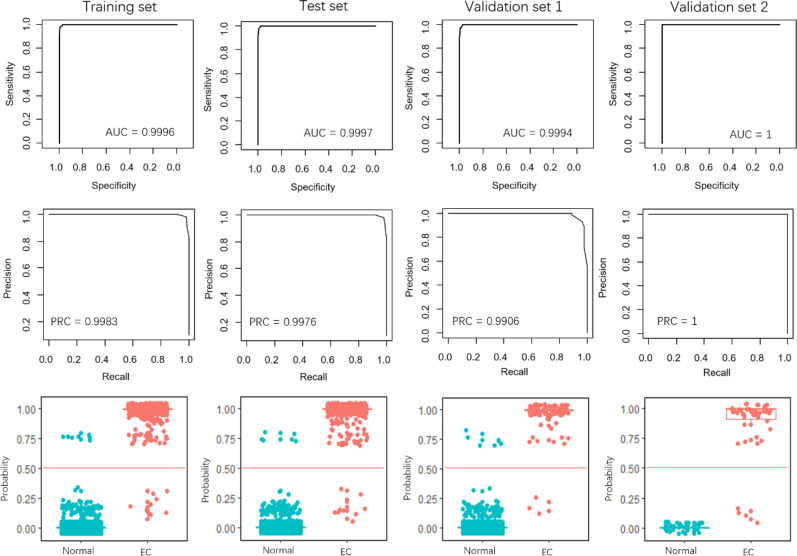
Performance evaluation of the 4-miRNA pair signature. The first row and the second row show the ROC and PRC curves for the training set, test set, and the two validation sets. The third row illustrates the prediction probability of the EC and normal samples in the four data sets.

The performance of the 4-miRNA pair signature was evaluated using the internal test set and two external validation sets. The 4-miRNA pair signature in these datasets yielded extremely high AUCs, all of them are close to 1 (**Figure 3**). Similar results also obtained for the PRCs, with scores higher than 0.99 in all datasets. The EC samples were clearly discriminated from the normal samples when the risk score threshold was 0.5 (**Figure 3**, lower panel). More importantly, iPAGE facilitated the decision of the classification threshold and only a few samples were uncorrected predicted.

**Figure 4:**
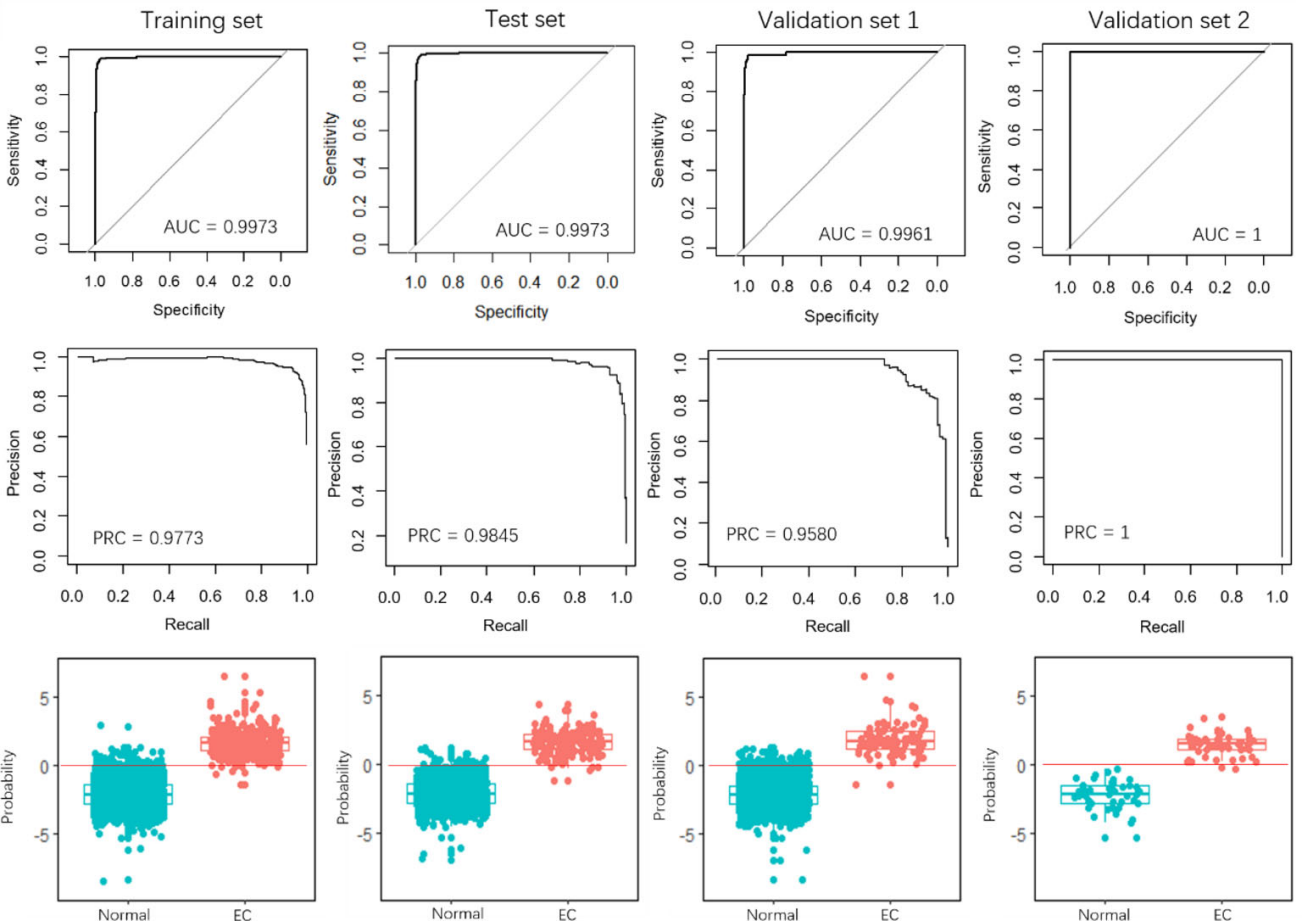
Performance evaluation of the 6-miRNA signature. The first row and the second row show the ROC and PRC curves for the training set, test set, and the two validation sets. The third row illustrates the prediction probability of the EC and normal samples in the four data sets.

Recently, Sudo et al. built an EC index using 6 serum miRNAs, *i.e.*, miR-8073, miR-6820-5p, miR-6794-5p, miR-3196, miR-744-5p, and miR-6799-5p, to accurately detect early-stage EC. Our results demonstrated that the 4-miRNA pair signature overall outperforms the 6-miRNA signature, especially in the validation set 1 (**Figure 4**). The AUCs of the 4-miRNA pair signature were over 0.9900, while the scores were around 0.9970 for the 6-miRNA signature in the four sets. Moreover, the PRCs of the 4-miRNA pair signature were more than 0.9990, whereas the scores were 0.9773, 0.9845, and 0.9580 for the 6-miRNA signature in the training set, test set, and validation set 1 (**Figures 3**, **4**).

**Figure 5:**
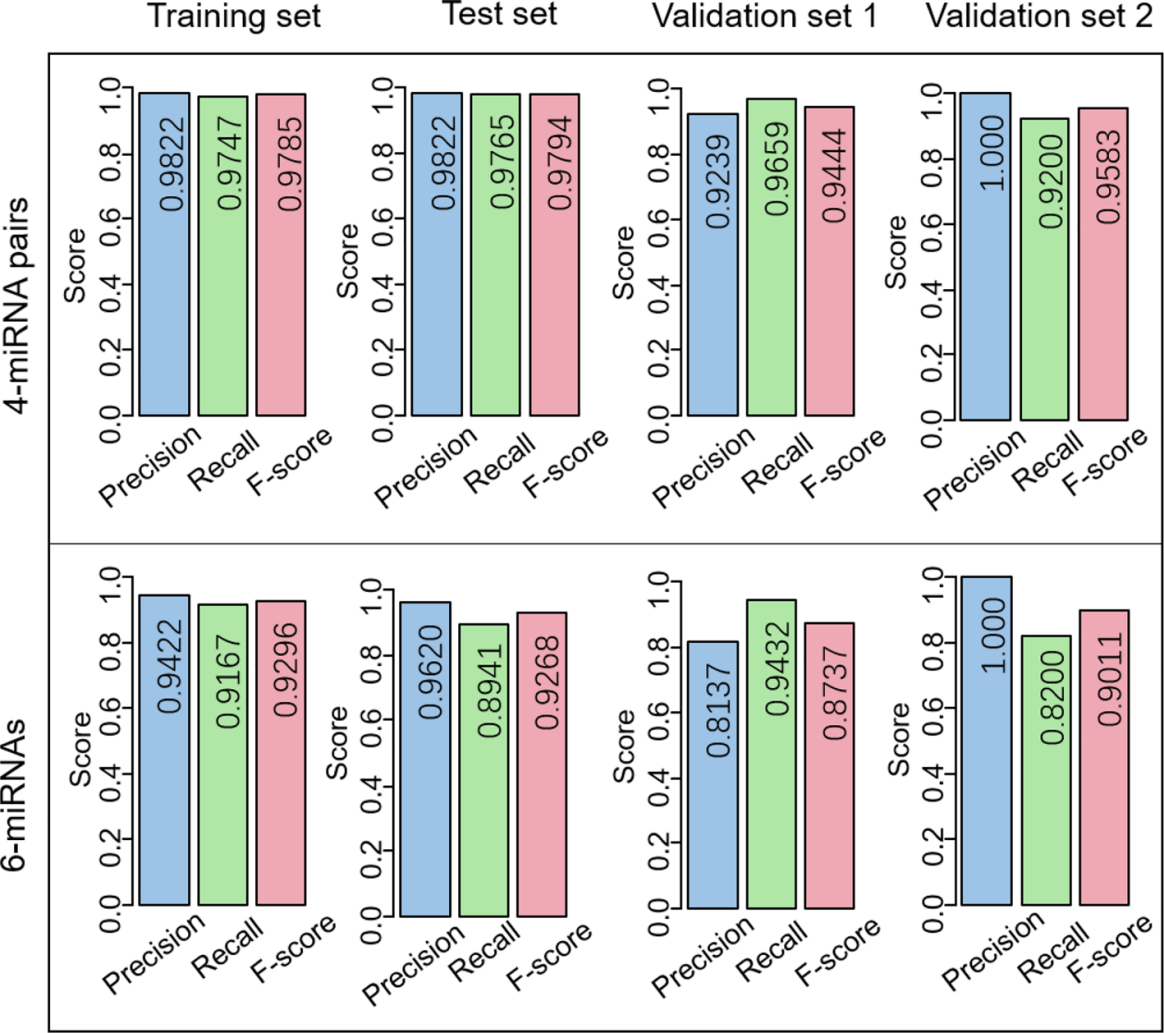
Comparison of the performance of the 4-miRNA pair signature and the 6-miRNA signature. Precision, recall, and F-score are used for evaluation.

More importantly, it is hard to determine a consistent threshold to predict whether a sample is EC or normal for the 6-miRNA signature, resulting in a low measurement of precision and recall. When the threshold was set 0, the 6-miRNA signature demonstrated the precision of 0.9422, 0.9620, and 0.8137 in the training set, test set, and validation set 1 (**Figure 5** and **Table 2**), respectively, while the scores were much higher for the 4-miRNA pair signature (0.9822, 0.9822, and 0.9239, respectively). The 6-miRNA signature yielded the recall of 0.9167, 0.8941, 0.9432, and 0.8200 in the training set, test set, validation set 1, and validation set 2, respectively, whereas the scores were improved to 0.9747, 0.9765, 0.9659, and 0.9200 for the 4-miRNA pair signature. The F-score of the 4-miRNA pair signature ranged from 0.9444 to 0.9794 in the four sets, which is consistently higher than that of the 6-miRNA signature (between 0.8737 and 0.9296).

**Table 2:** Evaluation of the performance of three miRNA signatures.

		Training set		Test set		Validation 1		Validation 2	
Signature	Reverse %	AUC	PRC	AUC	PRC	AUC	PRC	AUC	PRC
4-miRNA pairs	90%	0.9996	0.9983	0.9997	0.9976	0.9994	0.9906	1	1
5-miRNA pairs	95%	0.9994	0.9922	0.9995	0.9927	0.9994	0.9813	1	1
6-miRNAs		0.9973	0.9787	0.9962	0.9751	0.9961	0.9580	1	1

### The miRNA Pairs Are Associated With EC

In previous studies, miR-125b was reported to participate in tumor proliferation and cell cycle regulation as a suppressor regulator. Ma et al. identified a miRNA cluster including three miRNAs, *i.e.*, miR-99b, let-7e, and miR-125a, and observed the overexpression of the miRNAs in this cluster enhanced esophageal squamous cell carcinoma cell migration and invasion *in vitro* and induced an experimental metastasis *in vivo* (29). Wang et al. found that inhibition of miR-221 in 5-FU resistant cells resulted in reduced cell proliferation, increased apoptosis, restored chemosensitivity, and led to inactivation of the Wnt/β-catenin pathway mediated by regulating *DKK2* expression in esophageal adenocarcinoma (30). Mao et al. demonstrated that miR-1290 functions as a tumor oncogene by targeting *NFIX* to degrade its expression, which can promote proliferation, migration, and invasion during EC progression (31). The biological consequences that miR-1290 mediated by binding *NFIX* were also experimentally verified *in vitro*.

Other miRNAs such as miR-5100 and miR-6893 were also important regulators that are dysregulated in several types of cancers. The expression abundance of miR-5100 is associated with the prognosis of gastric cancer (32) and miR-6893 could restore circMTO1-regulated migration, invasion, and chemoresistance of cervical cancer cells (33). Therefore, the miRNAs in the miRNA pairs not characterized may serve as candidate regulators and therapy targets in the future clinical applications of EC.

## Discussion

We identified a 4-miRNA pair signature with the ability to diagnose patients with EC and validated its efficacy in two independent datasets. In total 8,378 samples were used to build and validate the diagnostic model. The signature demonstrated both AUCs and PRCs over 0.99 in all of the training set, test set, and two validation sets, which outperformed other state-of-art single miRNA signature. We also found literature supported evidences showing that the four miRNA pairs are highly associated with EC. Our results revealed that miRNAs pairs may serve as potential biomarkers for EC diagnosis.

Previously, we observed that using the expression value of lncRNAs or coding genes directly may lead to deviation, because high-throughput platforms are sensitive to various forms of technical variations (22, 34). Moreover, the generated continuous measurements were not measurable and comparable between different states due to the global biological alteration, even though they were preprocessed by plausible normalization methods (20, 21). iPAGE quantifies the relative expression of a pair of genes instead of the expression abundance of a single gene, which is an appropriate and sophisticated strategy to address the data preprocessing problem (22). Our results revealed that the relative expression is more reliable than the absolute expression value in the EC miRNA high-throughput data, which is an extension and approval of our previous discoveries. Recently, Liu et al. used 1,231 high-throughput miRNA-profiled serum samples to develop a diagnostic model for prostate cancer based on circulating miRNAs pairs and obtained approximate 0.99 for most of the measurements in a test and a validation set (35). This study also supported that circulating miRNA pairs are able to generate a robust diagnostic model in early diagnosis of cancers.

During the step of miRNA pair selection, we defined the reverse rate of 0.9 to filter miRNA pairs with a high ability to discriminate EC from the control samples. To assess the performance of iPAGE objectively, another threshold of 0.95 was also used to identify the reverse miRNA pairs. Using this threshold, 5-miRNA pairs were determined and it demonstrated AUCs and PRCs over 0.99 except the validation set 1, which yielded a PRC of 0.9813 (**Table 2**). Our findings revealed that iPAGE is a powerful tool for feature selection to reduce the data dimension and obtain relevant features for the machine learning models. In addition to the four miRNA pairs in miRPS consistently identified by running LASSO multiple times, the pairs selected by a majority of simulated calculations may also contribute to the classification. The four miRNA pairs were sufficient for diagnosis with a high accuracy, so it is not necessary to add the miRNA pairs less important into the penal. However, we may also consider these important pairs to improve the signature when it is not powerful enough.

In this study, the miRNA datasets used were all from the same platform of 3D-Gene Human miRNA V21_1.0.0, which limited the generalization of iPAGE and miRPS across different platforms. With the development of high-throughput technologies, an increasing number of miRNA datasets detected using different platforms will be available, more comprehensive cross-platform studies are warranted.

Our results revealed that circulating miRNAs pairs could serve as potential biomarkers for EC early diagnosis. iPAGE facilitates the steps of data preprocessing and feature selection, which is not only for lncRNA and mRNA data, but also for the miRNA expression data.

## Data Availability Statement

Publicly available datasets were analyzed in this study. This data can be found here: GSE122497, GSE106817, GSE112264.

## Author Contributions

LC and YS conceived of the idea. SZ and NZ prepared the data and analyzed the results. LC and YS supervised this work and wrote the manuscript. All authors contributed to the article and approved the submitted version.

## Funding

This work was supported by the Guangdong Basic and Applied Basic Research Foundation (2019A1515110097).

## Conflict of Interest

The authors declare that the research was conducted in the absence of any commercial or financial relationships that could be construed as a potential conflict of interest.

## Publisher’s Note

All claims expressed in this article are solely those of the authors and do not necessarily represent those of their affiliated organizations, or those of the publisher, the editors and the reviewers. Any product that may be evaluated in this article, or claim that may be made by its manufacturer, is not guaranteed or endorsed by the publisher.
